# Comparing voxel-based absorbed dosimetry methods in tumors, liver, lung, and at the liver-lung interface for ^90^Y microsphere selective internal radiation therapy

**DOI:** 10.1186/s40658-015-0119-y

**Published:** 2015-07-30

**Authors:** Justin K Mikell, Armeen Mahvash, Wendy Siman, Firas Mourtada, S Cheenu Kappadath

**Affiliations:** Department of Imaging Physics, The University of Texas MD Anderson Cancer Center, 1155 Pressler St, Unit 1352, Houston, TX 77030 USA; The University of Texas Graduate School of Biomedical Sciences at Houston, Houston, TX USA; Department of Interventional Radiology, The University of Texas MD Anderson Cancer Center, Houston, TX USA; Christiana Care Hospital, Newark, DE USA

**Keywords:** SIRT, Microspheres, SPECT, Dosimetry, Liver, Lung

## Abstract

**Background:**

To assess differences between four different voxel-based dosimetry methods (VBDM) for tumor, liver, and lung absorbed doses following ^90^Y microsphere selective internal radiation therapy (SIRT) based on ^90^Y bremsstrahlung SPECT/CT, a secondary objective was to estimate the sensitivity of liver and lung absorbed doses due to differences in organ segmentation near the liver-lung interface.

**Methods:**

Investigated VBDM were Monte Carlo (MC), soft-tissue kernel with density correction (SKD), soft-tissue kernel (SK), and local deposition (LD). Seventeen SIRT cases were analyzed. Mean absorbed doses ($\overline{AD}$) were calculated for tumor, non-tumoral liver (NL), and right lung (RL). Simulations with various SPECT spatial resolutions (FHWMs) and multiple lung shunt fractions (LSs) estimated the accuracy of VBDM at the liver-lung interface. Sensitivity of patient RL and NL $\overline{AD}$ on segmentation near the interface was assessed by excluding portions near the interface.

**Results:**

SKD, SK, and LD were within 5 % of MC for tumor and NL $\overline{AD}$. LD and SKD overestimated RL $\overline{AD}$ compared to MC on average by 17 and 20 %, respectively; SK underestimated RL $\overline{AD}$ on average by −60 %. Simulations (20 mm FWHM, 20 % LS) showed that SKD, LD, and MC were within 10 % of the truth deep (>39 mm) in the lung; SK significantly underestimated the absorbed dose deep in the lung by approximately −70 %. All VBDM were within 10 % of truth deep (>12 mm) in the liver. Excluding 1, 2, and 3 cm of RL near the interface changed the resulting RL $\overline{AD}$ by −22, −38, and −48 %, respectively, for all VBDM. An average change of −7 % in the NL $\overline{AD}$ was realized when excluding 3 cm of NL from the interface. $\overline{AD}$ was realized when excluding 3 cm of NL from the interface.

**Conclusions:**

SKD, SK, and LD are equivalent to MC for tumor and NL $\overline{AD}$. SK underestimates RL $\overline{AD}$ relative to MC whereas LD and SKD overestimate. RL $\overline{AD}$ is strongly influenced by the liver-lung interface.

## Background

Liver-directed selective internal radiation therapy (SIRT) has experienced clinical growth in recent years for the management of both hepatocellular carcinomas and metastatic disease from colorectal cancer, breast cancer, and neuro-endocrine tumors. Methods to calculate the administered activity for SIRT rely on body surface area and the assumption that activity is distributed uniformly throughout the liver, while the absorbed dose to the lung is based on the lung shunt fraction (LS) [1, 2]. A major limitation of these models is that they do not separate tumors from non-tumoral liver (NL) and are more accurately characterized as “safety planning” methods rather than “treatment planning.” The partition model [3] offers an improvement in that it separates tumors from NL, but it simplistically models all tumors as a single entity having a singular uptake fraction and assumes uniform activity distribution throughout the tumor and NL compartments.

Recent progress in post-therapy quantitative ^90^Y imaging with SPECT/CT and positron emission tomography/CT has facilitated voxel-level absorbed dose calculations. Voxel-based absorbed dose calculations are affected by the ^90^Y image quality in terms of quantitative accuracy and spatial resolution. Unlike other models, voxel-based absorbed dose calculations do not require tumor burden, tumor segmentation, or tumor uptake fraction as inputs for estimating absorbed dose at each voxel. Organ-at-risk and tumor segmentation are still necessary in voxel-based dosimetry, but segmentation is performed *to report* on the calculated absorbed doses and *not to explicitly calculate* the absorbed dose. Voxel-based dosimetry methods (VBDM) allow the absorbed dose calculation to be independent of the tumor and organ-at-risk segmentation.

There are several methods to calculate voxel-based absorbed doses for SIRT. However, little has been published in the literature comparing different VBDM, and the comparisons have been confined to the liver [4]. Lung dosimetry is of importance for SIRT because absorbed dose to the lung often limits the deliverable activity. The lung shunt fraction is estimated using 99mTc-macroaggregated albumin (MAA) with planar (or sometimes SPECT) imaging [5, 6]. In some instances, 99mTc-MAA SPECT/CT is performed to assess extra-hepatic uptake and these can in principle be used for therapy planning [7]. 99mTc-MAA SPECT scans have superior image quality compared to post-therapy bremsstrahlung ^90^Y SPECT scans, but there are studies showing MAA does not reliably predict the distribution of delivered ^90^Y microspheres [8]. To our knowledge, no previous study has reported the use of VBDM for determining absorbed dose to the lung and explored the implications of different VBDM in the liver-lung interface region [9, 10]. Both the EANM [5] and AAPM [6] provide guidance for clinical standard of practice ^90^Y microsphere therapy, but neither document addresses the effect of different VBDM which are under investigation.

In this study, we investigated differences among four VBDM for tumor, liver, and lung absorbed doses based on ^90^Y bremsstrahlung SPECT/CT imaging. Accuracy of the different methods at the liver-lung interface was estimated for different spatial resolutions and LS. Patient data was analyzed to determine the sensitivity of NL, right lung (RL), and total liver $\overline{AD}$ to the liver-lung interface.

## Methods

### Patient data

Patient data was processed to assess the impact of the different VBDM on absorbed dose calculations under realistic clinical situations. Accurate comparisons between dosimetry models can be achieved by using the same input patient data (administered activity and SPECT/CT images) into all of the VBDM. A total of 17 post-therapy ^90^Y SPECT/CT were selected for this study using a UT MD Anderson Cancer Center Institutional Review Board-approved retrospective chart review protocol where the informed consent requirement was waived. The mean administered activity was 2.81 ± 1.04 GBq (range 1.13–5.21 GBq). The administered activities were based on the package insert for the treatment device: ~120 Gy to treatment volume for glass microspheres. Adjustments were made to the activity based on the lung shunt fraction that was estimated by the 99mTc-MAA scans. Diagnostic CT or magnetic resonance images were manually registered to the SPECT/CT to aid in tumor delineation. A single interventional radiologist segmented the liver and tumors for all patients using the co-registered CT and/or magnetic resonance images. NL was generated by subtracting the tumor contours from the liver contour. RL was segmented using region growing in MIM Maestro v6.2 (MIM Software); RL was then inspected and manually adjusted by a physicist.

The ^90^Y SPECT/CT scans were acquired on a Symbia T16 (Siemens Medical Solutions) with medium-energy low-penetration collimation. SPECT data were acquired with a 90–125 keV primary window and 312–413 keV scatter window for 128 views over 360° with 28 s/view. A three-dimensional (3D) ordered-subset expectation maximization (Flash3D, Siemens Medical Solutions) SPECT reconstruction was performed using 4 iterations and 8 subsets with a 9.6 mm FWHM Gaussian post-filtering. The reconstructed isotropic voxel size was 4.8 mm. The reconstruction modeled geometric collimator response, CT-based attenuation correction using effective energy of the energy window width, and an energy window-based scatter correction [11]. The spatial resolution of the reconstruction was estimated to be 20 mm FWHM using an ^90^YCl_2_ line source in cold background.

Activity in each voxel (*A*_*ijk*_) was calculated by converting reconstructed SPECT counts to activity through a self-calibration factor defined as Administered Activity/Total Counts. We have assumed that all administered activities were within the SPECT field of view because most of the lung was included in the SPECT field of view; no correction for LS was applied. The Total Counts = ∑*C*_*ijk*_ where *C*_*ijk*_ represents the reconstructed counts in a voxel and the summation is over the entire SPECT volume.

Absorbed dose volume histograms of tumor, NL, and RL were generated for each patient and each VBDM. Correlations of $\overline{AD}$ from local deposition (LD), soft-tissue kernel (SK), and soft-tissue kernel with density correction (SKD) with Monte Carlo (MC) were investigated for tumor, NL, and RL. A qualitative evaluation of differences in the isodose distributions was also performed.

### Voxel-based dosimetry methods investigated: MC, SKD, SK, and LD

Four VBDM were investigated to calculate voxel-based absorbed doses for SIRT: MC, SKD, SK, and LD. MC was performed with the EGSnrc [12] user code DOSXYZnrc [13]. All electrons and photons were tracked down to kinetic energies of 1 keV. The simulation parameters included bound Compton scattering, Rayleigh scattering, atomic relaxations, Beithe-Heiler bremsstrahlung cross sections, simple bremsstrahlung angular sampling, spin effects, exact boundary crossing, and PRESTA-II. Voxel-level material (*M*_*ijk*_), activity (*A*_*ijk*_), and density (*ρ*_*ijk*_) distributions were derived from quantitative ^90^Y bremsstrahlung SPECT/CT. *ρ*_*ijk*_ was determined from the CT using a scanner- and technique-specific linear lookup table based on electron density phantom scans. *M*_*ijk*_ was generated by mapping *ρ*_*ijk*_ to one of four materials (air [14], lung [15], soft tissue [16], or bone [15]) based on density ranges.

We assume that ^90^Y microspheres have no biological clearance, so the total number of disintegrations in a voxel is given by ${N}_{ijk}={A}_{ijk}\cdot \frac{T_{1/2}}{ \ln (2)}$ where *T*_1/2_ is the physical half-life of ^90^Y (64.1 h). ^90^Y *β*^−^ has a maximum energy of 2.28 MeV corresponding to a maximum range of 11 mm in soft tissue[17], but the range increases to 44 mm in the lung with density 0.26 g/cc.

Table 1 summarizes the different VBDM investigated in this work. Absorbed doses calculated using MC are a function of material, total number of disintegrations, density, and the energy spectra of the beta particle emitted. Patient MC simulations were performed using 10^9^ histories. LD requires only the average energy of the beta particle and mass of each voxel. For SK and SKD, the absorbed dose soft tissue kernel was generated from MC simulations in an infinite soft-tissue medium with density of 1.04 g/cc using 2 × 10^9^ histories; it was validated by comparing with Lanconelli et al. [18]. The simulation yielded statistical uncertainty ≤0.002 % in the source voxel and ≤2.5 % at 40 mm. The kernel had isotropic voxel size of 4.8 mm matching the reconstructed SPECT. SK and SKD were calculated by convolving the total number of disintegrations with the kernel; convolutions were performed in IDL v8.2 (Exelis Visual Information Solutions). SKD was then scaled by the ratio of kernel density to voxel density [4].

**Table 1:** Characteristics of the different VBDM investigated

VBDM	Functional form	Notes
Monte Carlo (MC)	*F*(*M*_*ijk*_, *N*_*ijk*_, *ρ*_*ijk*_, *E*_90*Y*_)	*E*_90*Y*_ is the beta energy spectra per disintegration
Local deposition (LD)	${N}_{ijk}\cdot \frac{E_{avg}}{\rho_{ijk}\cdot \Delta V}$	*E*_*avg*_(0.937 *MeV*) is the average energy of the beta particle per disintegration. Δ*V* is the volume of a voxel.
Soft-tissue kernel (SK)	*N*_*ijk*_ ⊗ *K*_*i* ' *j* ' *k* '_	*K*_*i* ' *j* ' *k* '_ is obtained from a MC simulation of infinite uniform soft-tissue material with density of 1.04 g/cc.
Soft-tissue kernel with density correction (SKD)	$\left({N}_{ijk}\otimes {K}_{i^{\hbox{'}}{j}^{\hbox{'}}{k}^{\hbox{'}}}\right)\cdot \frac{1.04}{\rho_{ijk}}$	Assumes *ρ*_*ijk*_ is in units of g/cc

⊗ denotes convolution

### Assessing sensitivity of NL, RL, and total liver mean absorbed to the liver-lung interface

**Fig. 1:**
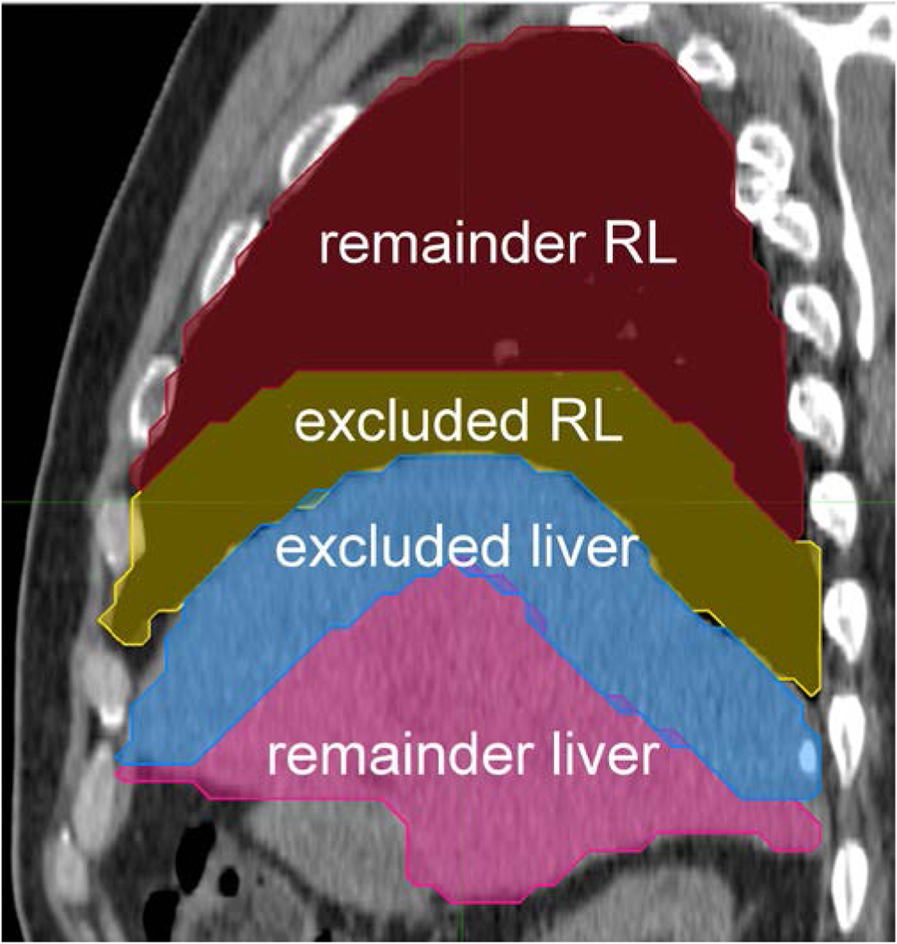
Sagittal view through liver and RL illustrating excluded regions from the liver-lung interface. Remainder RL (*red*), excluded RL (*yellow*), excluded liver (*blue*), and remainder liver (*pink*)

To assess sensitivity of NL, RL, and total liver $\overline{AD}$ to the liver-lung interface in patient data, we generated remainder VOI for total liver, NL, and RL by excluding regions extending 1, 2, or 3 cm from the liver-lung interface into both the liver and lung. The sensitivity of $\overline{AD}$ on segmentation was analyzed in Excel by plotting the $\overline{AD}$ to the original VOI as a function of the $\overline{AD}$ to the remainder VOIs and fitting a line to the data. Figure 1 shows an example of the remainder VOI generation.

### Simulation to estimate the impact of spatial resolution and LS on the accuracy of VBDM at the liver-lung interface

**Fig. 2:**
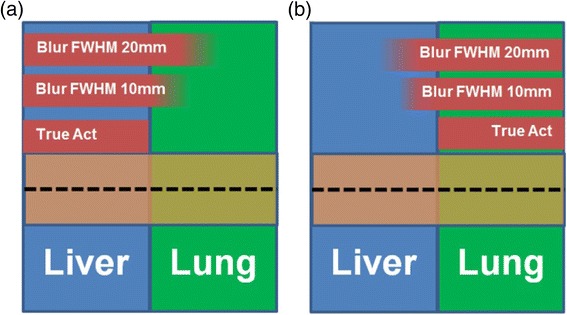
Schematic geometry of the simulations for the liver-lung interface with uniform activity in the slab representing either the liver (**a**) or lung (**b**). Arbitrary LS were achieved through superposition of individual VBDM for both liver and lung. Finite spatial resolution was modeled through Gaussian blurring. Data was averaged in the *orange region* to generate 1D absorbed dose profiles along the *dashed line*

Simulations were performed to estimate errors in the absorbed dose calculations around the liver-lung interface for the different VBDM as a function of spatial resolution and LS. We used a slab geometry with multiple spatial resolutions and LS; this simple simulation had two compartments (liver and lung) shown in Fig. 2 and does not use patient data. We placed a uniform amount of activity in the liver compartment representing a true activity distribution. To simulate limited spatial resolution, the activity in the liver was convolved with a Gaussian FWHM of 10 or 20 mm causing spill-out of the liver and spill-in to the lung. MC, SK, SKD, and LD voxel-level absorbed doses were then calculated on the three activity distributions (0, 10, 20 mm FWHM) and were normalized to the input activity. A similar process was carried out for the lung.

The density of the soft tissue and lung was set to 1.04 [16] and 0.26 g/cc [15], respectively. A newer ICRP report [19] lists the density of the lung as 0.25 g/cc, but we do not expect results to change significantly due to a 0.01 g/cc difference in density. The simulation volume consisted of 61 × 61 × 61 4.8 mm isotropic voxels. These voxels were padded such that the total dimensions were 200 × 200 × 200 cm^3^ approximating an infinite phantom. One-dimensional (1D) line profiles along the *z* axis were generated by averaging the central 7 × 7 voxels in each *x*-*y* plane.

For the three spatial resolutions, the liver and lung VBDM absorbed doses were combined via superposition by weighting the lung component by LS and the liver by 1-LS. We investigated LS of 1, 10, and 20 %. For quantitative comparison between VBDM, we defined the true absorbed dose distribution as the MC profile of 0 mm FWHM for a given LS; specifically, distance intervals along the 1D profile for which the different calculations agreed within ±10 % of the truth are reported.

## Results

### Comparing SKD, SK, and LD with MC for patients

**Fig. 3:**
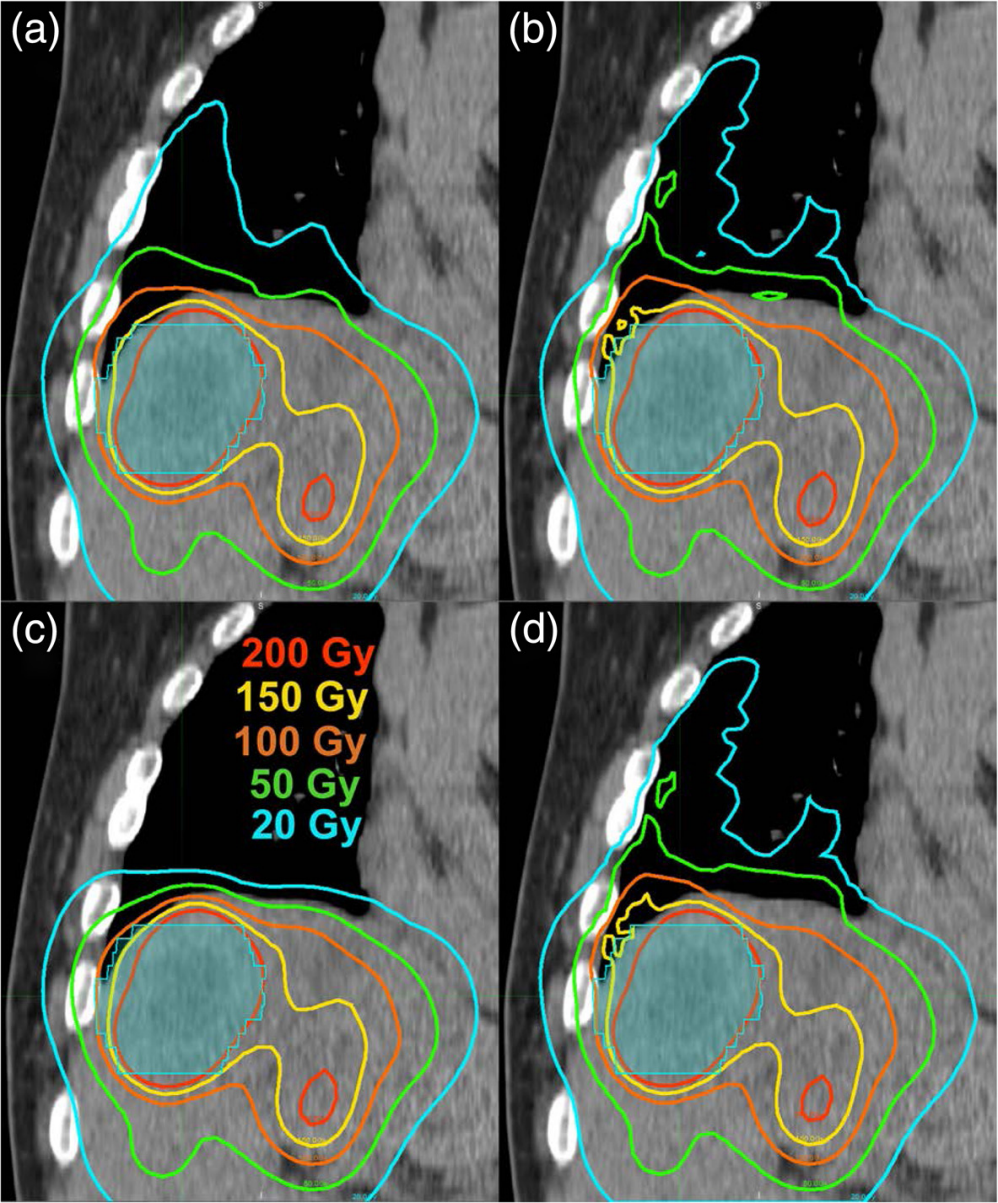
A coronal plane through the RL and liver illustrating salient differences between the four different VBDM: MC (**a**), LD (**b**), SK (**c**), SKD (**d**). The tumor (*shaded in cyan*) is 5.2 cm in length in the cranial-caudal direction

Figure 3 illustrates the salient differences in the apparent absorbed dose distribution stemming from the four VBDM; it shows the different absorbed dose calculations throughout the RL and liver on a coronal CT slice for a patient. The isodose curves deep within the liver were nearly identical for all four methods. The 20 Gy line extended furthest in the lung for SKD and LD followed by MC and then SK (least penetration into lung). The LD isodose distribution was very similar to the SKD distribution. There was an unequivocal qualitative difference in the lung absorbed dose distribution when SK was compared with MC, LD, or SKD, owing to the fact that SK assumes soft-tissue density of 1.04 g/cc regardless of the true density and material composition.

**Fig. 4:**
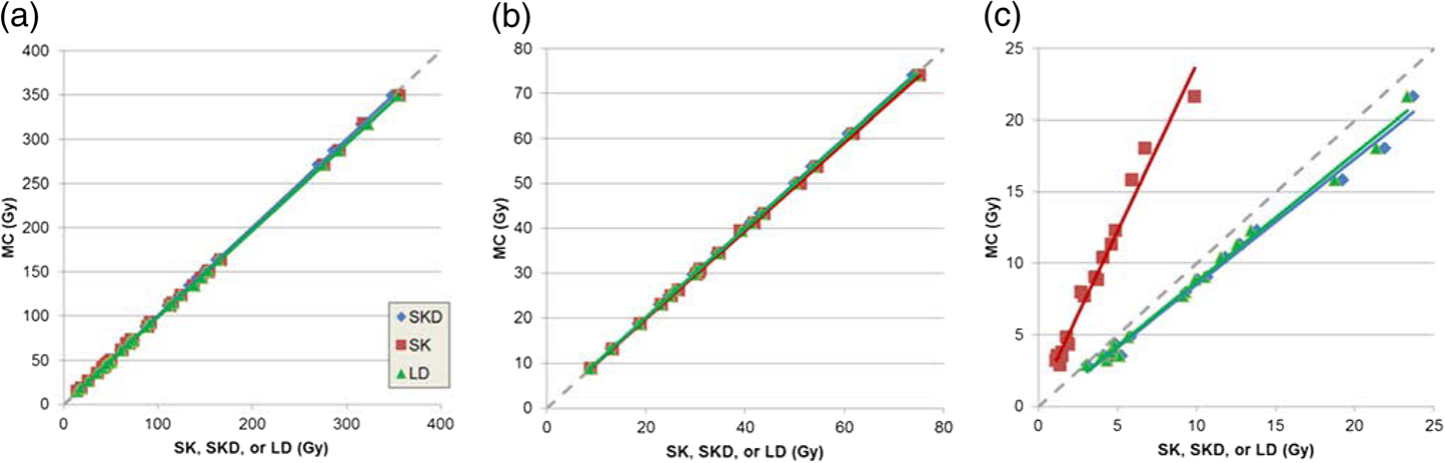
The correlation of patient absorbed doses from MC with those from LD (*green triangles*), SK (*red squares*), and SKD (*blue diamonds*) for tumor $\overline{AD}$ (*N* = 31) (**a**), NL $\overline{AD}$ (*N* = 17) (**b**), and RL $\overline{AD}$ (*N* = 17) (**c**) shown together with their linear fits. The *gray dashed line* represents the line of equivalence

The correlations in absorbed dose as estimated using LD, SK, and SKD in relation to the true values from MC are shown in Fig. 4. All the correlations had *R*^2^ > 0.975. Slopes of the fit lines ranged from 0.98 to 1.00 for tumors and NL. For RL $\overline{AD}$, the slopes were 0.88, 0.90, and 2.32 for SKD, LD, and SK, respectively. The summary of percent differences relative to MC are listed in Table 2. $\overline{AD}$ to tumors and NL using LD, SK, and SKD were within 5 % of MC . For $\overline{AD}$ to RL, LD had the best agreement (17 % on average) with MC, whereas SK had the poorest agreement (−60 % on average).

**Table 2:** Percent differences in $\overline{AD}$ when using SKD, SK, and LD compared with MC

	SKD vs. MC	SK vs. MC	LD vs. MC
Tumor $\overline{AD}$	−0.2 % ± 0.3 %,	1.6 % ± 1.2 %,	0.9 % ± 1.2 %,
	[−1.7 %, 0.0 %]	[−2.6 %, 3.1 %]	[−0.4 %, 4.7 %]
NL $\overline{AD}$	−0.3 % ± 0.1 %,	1.5 % ± 0.7 %,	−0.1 % ± 0.5 %,
	[−0.5 %, −0.1 %]	[−0.6 %, 2.3 %]	[−1.3 %, 0.6 %]
RL $\overline{AD}$	19.6 % ± 9.9 %,	−60.2 % ± 3.7 %,	17.4 % ± 9.4 %,
	[7.3 %, 48.3 %]	[−65.8 %, −52.7 %]	[6.5 %, 45.1 %]

*μ* ± *σ*, [min, max] of (100 × (calculation − MC)/MC)

### Sensitivity of total liver, NL, and RL mean doses to the liver-lung interface

**Fig. 5:**
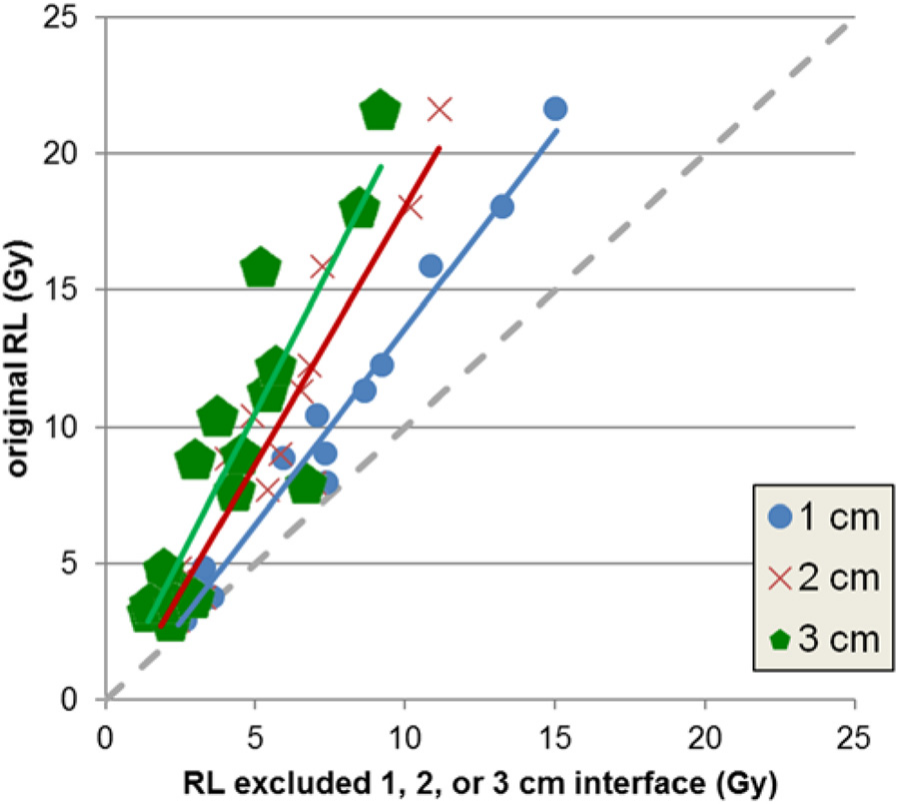
The MC $\overline{AD}$ to the patients’ RL (*N* = 17) when regions extending 1 (*blue circle*), 2 (*red x*), or 3 cm (*green pentagon*) from the liver-lung interface were excluded from the original RL VOI shown together with the linear fit. The *gray dashed line* represents the line of equivalence

Figure 5 shows the MC $\overline{AD}$ to the RL when regions extending 1, 2, or 3 cm from the liver-lung interface were excluded from both the liver and lung VOIs. The sensitivity was similar for all VBDM. For total liver, the slopes were 0.94, 0.87, and 0.74 when excluding 1, 2, and 3 cm from the interface, respectively; NL was less sensitive with slopes of 0.97, 0.94, and 0.92, respectively, and RL was the most sensitive with slopes of 1.43, 1.89, and 2.14, respectively. The RL $\overline{AD}$ sensitivity to the liver-lung interface was seen as a strong departure from the line of equivalence (Fig. 5). Excluding up to 3 cm of the liver-lung interface for the total liver and NL resulted in average differences of 4.1 and 6.9 %, respectively, from the original $\overline{AD}$ to VOIs (without excluded regions), suggesting relative insensitivity to the interface region. On the contrary, excluding up to 3 cm of the interface for the RL led to an average difference of −48.4 % from the original $\overline{AD}$, suggesting that RL $\overline{AD}$ is very sensitive to the interface region.

### Effect of spatial resolution and LS on accuracy of the VBDM at the liver-lung interface

**Fig. 6:**
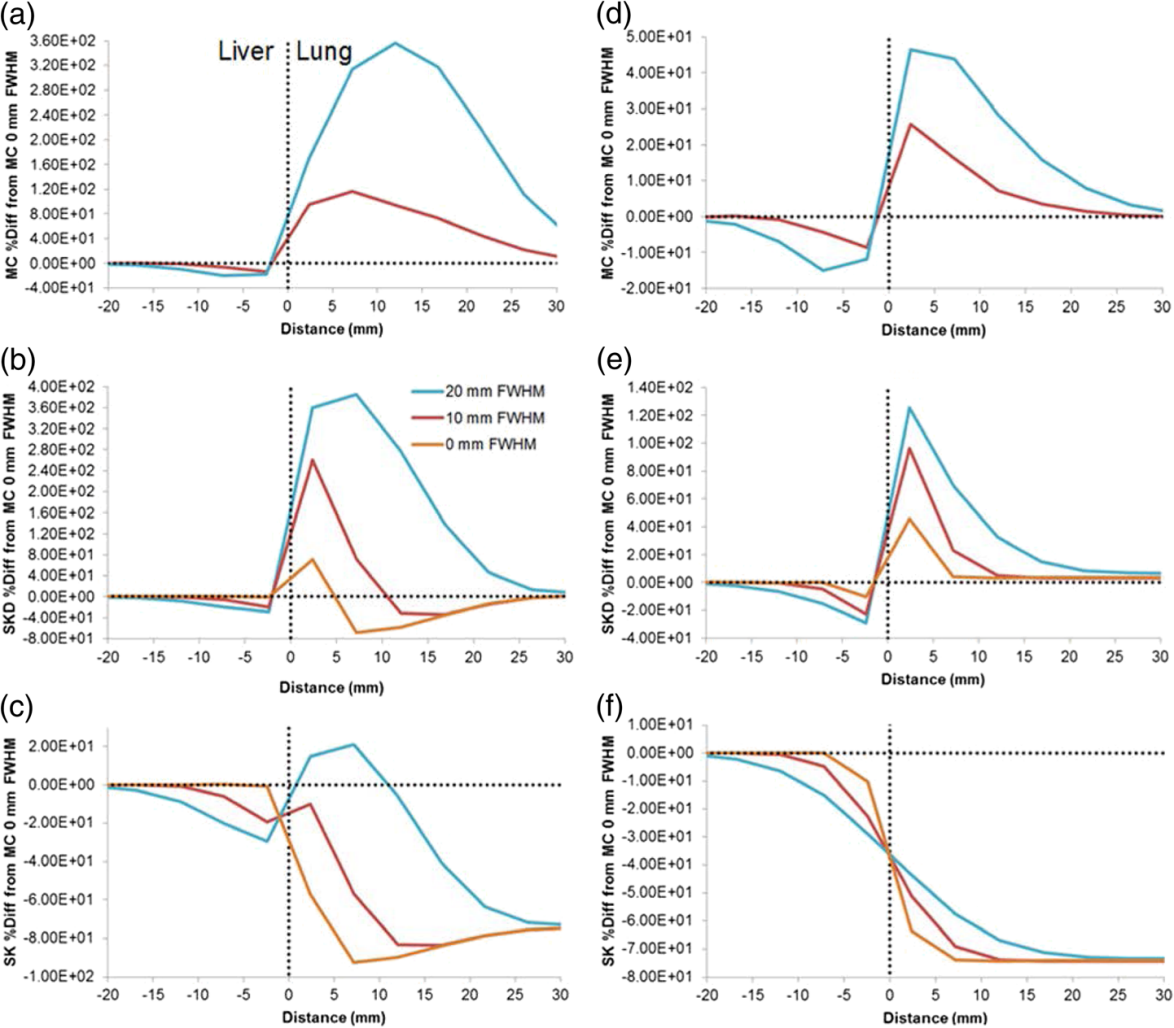
1D profiles from VBDM simulations with different spatial resolution (20 mm FWHM (*blue*), 10 mm FWHM (*red*), 0 mm FWHM (*orange*)) at the liver-lung interface showing percentage differences from MC without blurring. LD is omitted since it is similar to SKD. LS is 1 % in MC (**a**), SKD (**b**), and SK (**c**). LS is 20 % in MC (**d**), SKD (**e**), and SK (**f**).

Figure 6 shows the percent differences of the different absorbed dose calculations relative to the truth (MC of true activity distributions) for different LS and different FWHM. LD is not displayed since the differences are similar to SKD. Errors on the liver side of the interface were generally within 30 % and approached 0 as the blurring decreased to 0 and moved away from the interface deeper into the liver. Near the lung interface, errors for 20 mm FWHM blurring and 1 % LS were within 20 % when using SK compared to errors over 200 % for MC and SKD. Table 3 lists the distance intervals where agreement with MC was within 10 %; on the liver side, agreement to within 10 % for all methods was found beyond 4, 6, and 12 mm from the interface for 0, 10, and 20 mm FWHM blurring, respectively, with a LS from 1 to 20 %. For MC, LD, and SKD in the lung, agreement was found beyond 26, 31, and 39 mm for 0, 10, and 20 mm FWHM blurring, respectively.

**Table 3:** Intervals in millimeters where the VBDM are accurate to within 10 % as a function of LS and FWHM where positions ≤0 represents the liver, positions >0 represents the lung, and 0 represents the liver-lung interface

VBDM	LS (%)	Blurring FWHM (mm)		
		0	10	20
MC	1	(−∞, ∞ )	(−∞,−4) U (31,∞)	(−∞,−12) U (39, ∞)
	10	(−∞, ∞ )	(−∞, −3) U (−2, −1) U (17, ∞)	(−∞ ,−11) U (−2,−1) U (26, ∞)
	20	(−∞, ∞ )	(−∞, 0) U (11, ∞)	(−∞, −10) U (−2,−1) U (21,∞ )
LD	1	(−∞, −4) U (−2,−1) U (26, ∞)	(−∞,−4) U (−2,−1) U (7, 8) U(26, ∞)	(−∞,−11) U (−2,−1) U (24, ∞)
	10	(−∞, 0) U (11,∞)	(−∞, −5) U (−2,−1) U (7, ∞)	(−∞,−11) U (−2,−1) U (19, ∞)
	20	(−∞, ∞)	(−∞ ,−5) U (−2,−1) U (8,∞)	(∞,−9) U (−2,−1) U (16, ∞ )
SKD	1	(−∞,−2) U (4,5) U (23,∞)	(−∞,−6) U (−2,−1) U (10,11) U (23, ∞)	(−∞, −11) U (−2,−1) U (29,∞)
	10	(−∞, −1) U (6, ∞)	(−∞,−6) U(−2,−1) U(11,∞)	(−∞,−11) U (−2,−1) U (23,∞)
	20	(−∞, −1) U (7,∞)	(−∞, −6) U (−2,−1) U (11, ∞)	(−∞,−10) U (−2,−1) U (21, ∞)
SK	1	(−∞, −2)	(−∞, −6)	(−∞, −11) U (−0.5, 2) U (9, 13)
	10	(−∞, −2)	(−∞, −6)	(−∞, −11)
	20	(−∞, −2)	(−∞, −6)	(−∞, −10)

The true absorbed dose distribution was MC with FHWM = 0. We employed interval notation (e.g., (*x*1, *x*2) ∪ (*x*3, *x*4)

SK approximated the true absorbed dose near the lung interface well for 1 % LS with blurring of 10 and 20 mm but significantly underestimated the absorbed dose at the lung interface and deep into the lung for the higher LS. MC matched the true lung absorbed dose better for the higher LS and lower blurring. SKD and LD overestimated near the lung interface compared to MC, but they both approached the true value deep (>39 mm) within lung.

LD, SK, SKD, and MC approached the same value deep (>12 mm) within the liver, and they were all similar on the liver side of the interface, with MC performing slightly better than the others given a larger FWHM. On the liver side of the interface, LD, SK, and SKD all underestimated the absorbed dose similarly when the activity distribution was blurred.

**Fig. 7:**
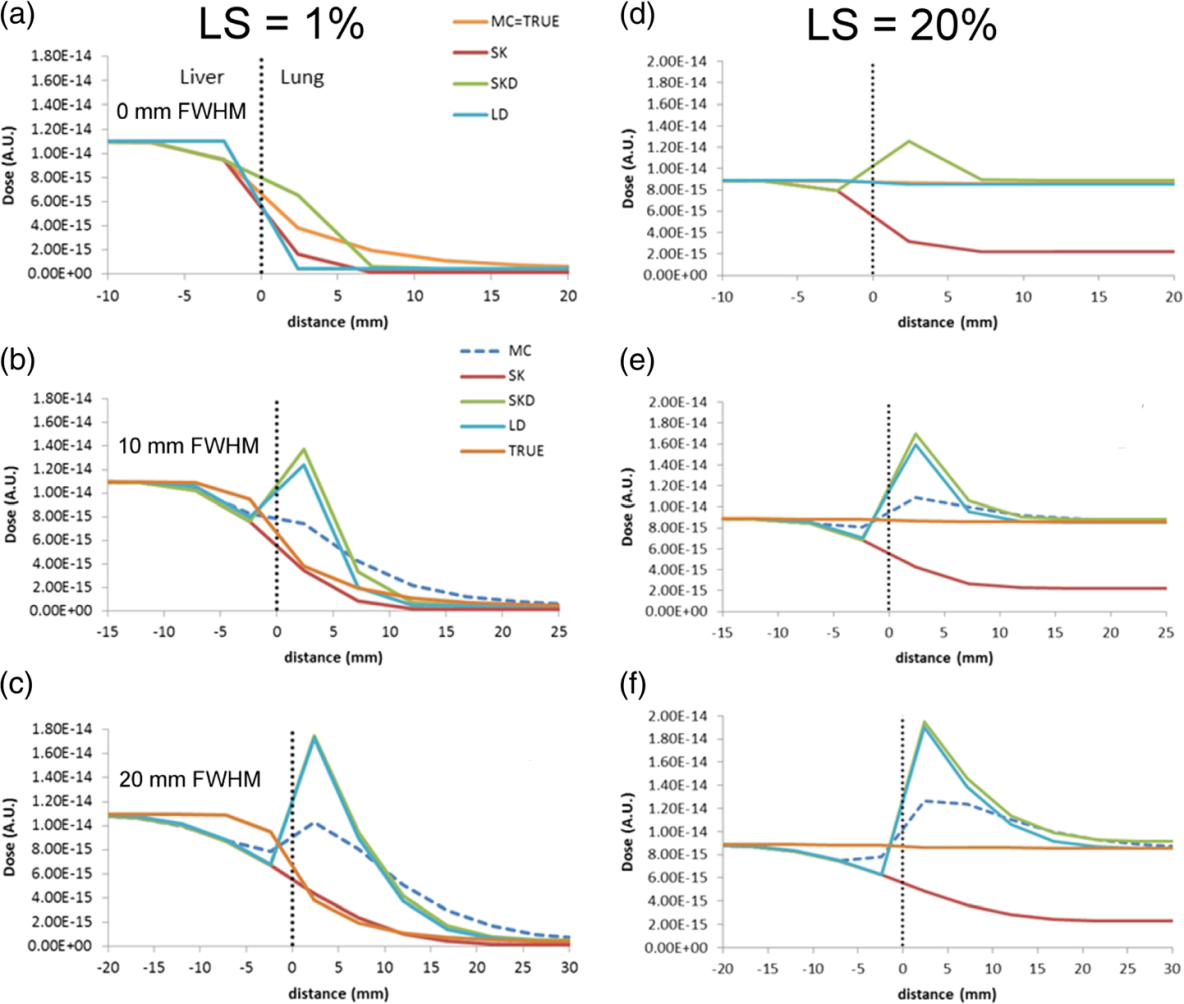
1D dose distributions at the liver-lung interface to compare the four VBDM for different spatial resolution and LS. LS is 1 % in 0 mm FWHM (**a**), 10 mm FWHM (**b**), and 20 mm FWHM (**c**) while LS is 20 % in 0 mm FWHM (**d**), 10 mm FWHM (**e**), and 20 mm FWHM (**f**). *A.U.* arbitrary units

Figure 7 provides context of the relative differences in Fig. 6 by showing line profiles of absorbed dose in arbitrary units for the VBDM with different lung shunt fractions and spatial resolutions. Figure 7 can be used to estimate absolute errors in the absorbed dose near the interface. For example, if one assumes the absorbed dose within the liver far from the interface is 80 Gy, then the SKD absorbed dose in the lung at 7 mm from the interface for LS = 1 % and FWHM = 20 mm would be ≈9.4E-15/1.1E-14 × 80 Gy ≈ 68 Gy whereas the true value would be ≈1.9E-15/1.1E-14 × 80 Gy = 14 Gy.

## Discussion

Our NL and tumor absorbed dose results are similar to those reported by Dieudonne et al. [4]; both studies showed better agreement with MC when using SKD instead of SK. Our work adds to the body of knowledge on ^90^Y dosimetry in part by assessing absorbed dose differences in the patient data due to differences in methodology. We also included LD voxel-based estimates in our comparison; these estimates are relevant since investigators have recently begun using LD in voxel-based absorbed dose calculations following SIRT [20, 21]. Lung dosimetry is also of central importance in SIRT because lung absorbed dose limits the administered activity of ^90^Y and can prevent patients from receiving adequate therapeutic tumor absorbed doses. However, to date, no work has compared VBDM for the lung and in the liver-lung interface based on ^90^Y bremsstrahlung SPECT/CT imaging.

The correlations of LD, SK, and SKD with MC for mean absorbed dose to tumors, NL, and RL could potentially be used to convert mean absorbed doses between the different VBDM for our image acquisition protocol.

The sensitivity of the patients’ RL, NL, and total liver $\overline{AD}$ to the liver-lung interface agrees with the trend that larger distances from the interface are required in the lung (relative to the liver) to reach accurate absorbed doses. The RL $\overline{AD}$ decreased by ~50 % (8.8 ± 5.4 Gy to 4.2 ± 2.3 Gy) when 3 cm of the interface was excluded, whereas the total liver $\overline{AD}$ only increased by ~3 % (45.1 ± 12.7 Gy to 46.6 ± 15.0 Gy) for a similar interface exclusion. From a clinical perspective, this finding highlights that the liver $\overline{AD}$ is not sensitive to the interface region, but RL $\overline{AD}$ is sensitive to the interface and the community needs to establish standards and guidelines for lung segmentation to ensure proper reporting of lung absorbed doses when using VBDM. These findings call for careful consideration of lung dose based on post-therapy ^90^Y imaging (and to a lesser degree pre-therapy 99mTc-MAA) for cumulative lung dose calculation as part of repeat treatments where cumulative lung dose is not to exceed 50 Gy. Patient respiratory motion further degrades the effective spatial resolution at the liver-lung interface because motion correction techniques are not available in commercial SPECT/CT systems.

The simulated estimates of accuracy for MC, LD, SK, and SKD around the liver-lung interface as a function of LS and spatial resolution FWHM showed that all four VBDM investigated are within 10 % of the true liver absorbed dose when deeper than 12 mm from the liver-lung interface; this distance is expected to increase for larger FWHM and lower LS. Using MC, LD, or SKD, a similar accuracy was achieved in the lung when deeper than 39 mm from the interface. SK is not suitable for estimating accurate deep lung absorbed doses, but in the special case where LS is small and FWHM is large, SK may provide accurate estimates in close proximity to the liver-lung interface; this transient accuracy occurs due to SK errors in lung dosimetry canceling errors due to spill-in/out at the interface.

For the clinical results (tumors, NL, and RL), we have only investigated differences among VBDM in this work. Although we estimated accuracy of VBDM at the liver-lung interface through simulations, we have not performed such simulations for patient data. Future work should include the use of virtual phantoms where the true activity distribution is known followed by imaging simulation and application of VBDM to estimate true accuracy of such methods in patients.

Some have argued that LD may be preferable to transport (SK, SKD, or MC) for pure-beta emitters such as ^90^Y [22]; radiation transport spreads ^90^Y beta energy deposition locally at ~5 mm scale in soft tissue. Their rationale is that the finite spatial resolution of the imaging system (typically >10 mm in emission imaging) can account for beta radiation transport. However, realistic particle transport will depend on tissue type and density (e.g., soft tissue vs. lung). Although not discussed here, the collapsed cone convolution is another VBDM that is accurate at the lung soft-tissue interface for SIRT [23].

Our results on accuracy suggest that if one uses VBDM, then to reduce errors in absorbed dose estimates at the interface, the effective spatial resolution (physical spatial resolution and motion blurring) at the liver-lung interface should be minimized. Improvements in SPECT image quality would provide improved voxel-based activity distribution, especially at the liver-lung interface.

One limitation of our study stems from the use of a free-breathing CT scan as part of SPECT/CT. Consequently, the contoured liver/lung interface could be from any point of the respiratory cycle. In the analysis of the interface patient data, the results must be viewed critically since there is not a straightforward method to determine the correct spatial location or a reference volume for the lung. We have only estimated errors in 1D absorbed doses for misplaced activity at the lung-liver interface due to effective spatial resolution, not the change in activity due to incorrect attenuation correction at the interface. Future work could involve analysis with some respiratory motion management such as breath hold, average CT, or 4D-CT. There was also uncertainty in the delineation of the tumor, liver, and lung and registration errors between the diagnostic contrast examination and the attenuation scan on the SPECT. Our RL segmentation methodology was similar to Busse et al. who reported that region growing resulted in an average error of 7 % for lung mass estimates based on free-breathing CT scans of the thorax [24]. We would like to point out that the patient data analysis was based on a single SPECT/CT model and customized imaging protocol and segmentation by a single physician using data from our institution.

There are limitations to all imaging acquisition and reconstruction protocols. Differences at the liver-lung interface depend on several parameters including spatial resolution, respiratory motion, activity distribution near the interface, free-breathing CT vs average CT vs breath-hold CT, and the corresponding scatter and attenuation compensations during reconstruction. Consequently, the magnitude of the sensitivity of right lung, total liver, and non-tumoral liver absorbed dose to the liver-lung interface may change if PET/CT or a different SPECT/CT acquisition protocol or reconstruction algorithm such as Rong et al. [25] is used. In this work, we have investigated differences among four VBDM for tumor, liver, and lung absorbed doses based on a given ^90^Y bremsstrahlung SPECT/CT imaging; the magnitude of the clinical findings in this work may change with different acquisition or reconstruction protocols, but the trends in sensitivity to the interface should hold. Thus, these findings are not restricted to any one specific ^90^Y image generation technique.

SPECT calibration is important for reconstructing quantitative images. We estimated the 95 % confidence interval in our self-calibration to be ~10 %, based on 25 different patient scans. The purpose of this work was to investigate differences between VBDM, and thus by design the administered activity and total SPECT counts were the same between different VBDM. Therefore, the results of this work are not sensitive to the uncertainties in SPECT self-calibration.

## Conclusions

Voxel-based dosimetry was performed using post-therapy ^90^Y bremsstrahlung SPECT/CT. Multiple VBDM (MC, LD, SKD, SK) were investigated and compared to MC for $\overline{AD}$ for tumor, NL, and RL. Differences were equivalent (<5 %) for tumor and NL $\overline{AD}$, with SKD agreeing best with MC. Larger differences were found for the RL $\overline{AD}$, with LD agreeing best with MC and SK producing dramatically incorrect values deep in the lung. Simulations of the liver-lung interface for multiple effective spatial resolutions and LSs were used to estimate nominal distance from the liver-lung interface where good accuracy was achieved deep within the liver and deep within the lung. Finite spatial resolution was shown to cause RL $\overline{AD}$ estimates to be sensitive to the liver-lung interface region.

## Abbreviations

VBDM: voxel-based dosimetry methods
MC: Monte Carlo
SK: soft-tissue kernel
SKD: soft-tissue kernel with density correction
LD: local deposition
NL: non-tumoral liver
RL: right lung
LS: lung shunt fraction
$\overline{AD}$: mean absorbed dose
SIRT: selective internal radiation therapy
SPECT: single-photon emission computed tomography
CT: computed tomography
FWHM: full width at half maximum
VOI: volume of interest
